# PTEN Regulates BCRP/ABCG2 and the Side Population through the PI3K/Akt Pathway in Chronic Myeloid Leukemia

**DOI:** 10.1371/journal.pone.0088298

**Published:** 2014-03-06

**Authors:** Fang-Fang Huang, Li Zhang, Deng-Shu Wu, Xiao-Yu Yuan, Fang-Ping Chen, Hui Zeng, Yan-Hui Yu, Xie-Lan Zhao

**Affiliations:** 1 Department of Hematology, Xiang Ya Hospital, Central South University, Changsha, Hunan, China; 2 Department of Hematology, West China Hospital, Si Chuan University, Chengdu, Sichuan, China; UNIVERSITY MAGNA GRAECIA, Italy

## Abstract

A small population of cancer stem cells named the “side population” (SP) has been demonstrated to be responsible for the persistence of many solid tumors. However, the role of the SP in leukemic pathogenesis remains controversial. The resistance of leukemic stem cells to targeted therapies, such as tyrosine kinase inhibitors (TKIs), results in therapeutic failure or refractory/relapsed disease in chronic myeloid leukemia (CML). The drug pump, ATP-binding cassette sub-family G member 2 (ABCG2), is well known as a specific marker of the SP and could be controlled by several pathways, including the PI3K/Akt pathway. Our data demonstrated that compared with wild-type K562 cells, the higher percentage of ABCG2+ cells corresponded to the higher SP fraction in K562/ABCG2 (ABCG2 overexpressing) and K562/IMR (resistance to imatinib) cells, which exhibited enhanced drug resistance along with downregulated phosphatase and tensin homologue deleted on chromosome -10 (PTEN) and activated phosphorylated-Akt (p-Akt). PTEN and p-Akt downregulation could be abrogated by both the PI3K inhibitor LY294002 and the mTOR inhibitor rapamycin. Moreover, in CML patients in the accelerated phase/blastic phase (AP/BP), increased SP phenotype rather than ABCG2 expression was accompanied by the loss of PTEN protein and the up-regulation of p-Akt expression. These results suggested that the expression of ABCG2 and the SP may be regulated by PTEN through the PI3K/Akt pathway, which would be a potentially effective strategy for targeting CML stem cells.

## Introduction

Chronic myeloid leukemia (CML) is a clonal bone marrow stem cell disorder that accounts for 7–20% of all leukemia cases and has an estimated incidence of 1–2 per 100,000 worldwide [1]. CML arises by a reciprocal translocation between the long arms of chromosome 9 and chromosome 22 in an early hematopoietic stem cell (HSC) to produce the Philadelphia chromosome [2], [3], [4]. Although tyrosine kinase inhibitors (TKI) such as imatinib mesylate, nilotinib and dasatinib have been proven to be highly effective in the treatment of CML [5], [6], [7], a considerable number of the patients unfortunately face relapse or are unable to obtain complete remission during TKIs therapy [8],[9],[10]. The relative quiescence of CML stem cells or the overexpression of drug transporters are currently considered the main factors contributing to impaired effectiveness for CML treatments [11], [12], [13].

The side population (SP), which can be identified and sorted by the efflux of Hoechst 33342, expresses stem cell properties, such as pluripotency and differentiation ability. ATP-binding cassette sub-family G member 2 (ABCG2), which is also known as breast cancer resistance protein (BCRP), is defined as a specific marker of the SP in a variety types of stem cells based on its ability to efflux Hoechst 33342 [14], . Previous results from adult acute myeloid leukemia demonstrated that SP cells may represent candidate leukemia stem cells. However, the role of ABCG2 expression and the SP phenotype in the mechanism of resistance to TKI in CML stem cells remains unclear [17]. Interestingly, the tumor suppressor gene phosphatase and tensin homologue deleted on chromosome-10 (PTEN), which is often deleted or inactivated in many solid tumor types [18], [19], [20], has also been shown to be down-regulated by BCR-ABL in CML stem cells, and its deletion can accelerate CML development through the regulation of its downstream target, Akt1 [21]. Moreover, PTEN was described as regulating the SP but not the expression of ABCG2 in glioma tumor stem-like cells through the PI3K/Akt pathway [22]. We speculate that the crosstalk between ABCG2 and PTEN in CML mediates therapeutic resistance and disease progression in CML cells, particularly within the SP compartment. As such, we analyzed data from both CML cell lines and clinical samples from CML patients (Tab. 1).

**Table 1:** Characteristics of patients with CML (n = 96).

Stage of CML	Total no. of Pts	M/F	Age, median (range) [years]
**CML-CP**	61	32/29	40/(21–61)
**CML-AP/BP**	35	15/22	42/(24–65)

## Materials and Methods

### Cell lines and culture condition

K562 cells were purchased from a cell resource center (Xiang-Ya Medical College, Central South University, Hunan, China). K562/IMR and K562/AO2 cells were kindly obtained from the Institute of Hematology and Blood Diseases Hospital (Chinese Academy of Medical Sciences and Peking Union Medical College, Tianjin, China) and the First Affiliated Hospital of Zhengzhou University (Zhengzhou, China), respectively. Cell lines were routinely maintained in RPMI-1640 medium (GIBCO, NY, USA) supplemented with 10% fetal bovine serum (FBS; HyClone, MA, USA) and 1% penicillin/streptomycin (Sigma, MO, USA) in the humidified atmosphere of a 5% CO_2_ incubator at 37°C. The PI3K inhibitor LY294002 (Invitrogen, Carlsbad, CA, USA) and the mTOR inhibitor rapamycin (Invitrogen, Carlsbad, CA, USA) were added to leukemia cells for 72 hours prior to mitoxantrone in some experiments.

### Patient characteristics

From 2010 to 2012, bone marrow samples were obtained from 96 CML patients and 10 healthy candidate donors for hematopoietic stem cell transplantation as controls enrolled at the Xiang-Ya Hospital of Central South University, Hunan, China (Table 1). All patients and donors gave informed consent. The protocol was approved by the Medical Ethic committee of Xiangya Hospital, Central South University. Participants provided their written informed consent to participate in this study. The diagnosis and classification of the leukemia were based on 2008 World Health Organization's criteria. Mononuclear cells (MNCs) were obtained by density centrifugation over Ficoll-Paque (Sigma, St Louis, MO, USA) and stored at −80°C.

### Cytotoxicity assay

Cells were cultured with various concentrations of the indicated agents. Cell viability was determined by a CCK8 assay (Nan Jing Key Gen Biotechnology, Nan Jing, China). Briefly, cells were seeded in 96-well culture plates (8×10^3^ per well) in 100 µL media for 12 h. Subsequently, different concentrations of mitoxantrone (0.01–1.0 µM) were added to the wells and incubated for 72 h. At the end of the treatment, 10 µM of CCK8 solution was added to each well for 1 h culture at 37°C. Absorbance was measured with a spectrophotometer (Thermo Scientific Evolution 600, China) at a wavelength of 450 nm and compared with 630 nm.

### Apoptosis assessment

After a 48-h culture, at least 1×10^5^ untreated and mitoxantrone-treated cells were collected and washed twice with cold PBS, stained with 5 µl of Annexin V-FITC and 5 µl of propidium iodide (PI) for 15 min, and subjected to flow cytometry (Becton Dickinson, CA, USA) to analyze apoptosis.

### Lentiviral infection of cell lines

The lentiviral constructs PsPAX2, VSVG, pSIN4-EF2-ABCG2-IRES-Neo (from Dr. Ren-He Xu's laboratory) and pSIN4-EF2-EGFP-IRES-Neo plasmids (from Dr. James Thomson's laboratory) were used to make viral stocks by transfection of 293FT cells using Lipofectamine 2000 (Invitrogen, Carlsbad, CA, USA), as previously described. The lentiviral supernatants were harvested 72 h post-transfection and were filtered (0.45 µM) prior to infection of the cell lines. After a 48-h infection (MOI: 5–50), the K562 cell lines were allowed to recover for 24 h in fresh media and were thereafter referred to K562/ABCG2 cells. The cells were then allowed to grow for 72 h before being subjected to additional assays.

### Western blot analysis

Protein (50 µg/sample) was separated in 8% sodium dodecyl sulfate-polyacrylamide gel electrophoresis (SDS-PAGE) and then transferred electrophoretically to polyvinylidene fluoride (PVDF) membranes. The membranes were saturated in PBS-T containing 5% nonfat milk (blocking buffer) overnight at 4°C with the primary antibodies. The dilutions of the antibodies used for western blotting were as follows: PI3K p85 (Tyr458)/p55 (Tyr199) 1∶1000 (Cell Signaling Technology, Beverly, MA, USA), PI3K 1∶1000 (Abcam, MO, USA), phosphorylated-Akt (p-Akt) (S473) 1∶400 (Cell Signaling Technology, Beverly, MA, USA), PTEN 1∶200 (Abcam, MO, USA) and β-actin 1∶1000 (Abcam, MO, USA). The membranes were then incubated for 60 min at room temperature with an HRP-linked secondary antibody (Santa Cruz Biotechnology, Santa Cruz, CA, USA). Protein bands were visualized using the Chei Doc™MP imaging system (BioRad). The blots shown are representative of three different experiments.

### RNA isolation, RT-PCR and real-time RT-PCR

Total RNA (2 µg) was reverse transcribed using the M-MLV First Strand Kit (Invitrogen, Carlsbad, CA, USA). The transcript levels for the genes of interest were normalized to the GAPDH transcripts. The gene-specific primers and RT-PCR conditions are summarized in Table 2. Real-time PCR was performed using a SYBR qPCR Mix (Toyobo, TOYOBO CO., LTD, JAPAN) on an ABI StepOnePlus (Applied Biosystems, Foster City, CA, USA) with the specific primers. The thermal cycler conditions were as follows: 95°C for 1 min, followed by 40 cycles of 95°C for 10 s, 61°C for 20 s, and 72°C for 50 s.

**Table 2:** PCR primers and conditions.

Gene	Strand	Primer sequences	Annealing temperature
**PTEN**	sense	5′-ACCAGGACCAGAGGAAACCT-3′	61.5°C
	anti-sense	5′-GCT AGCCTCTGGATTTGACG-3′	
**ABCG2**	sense	5′-ATTGAAGGCAAAGGCAGATG-3′	61.0°C
	anti-sense	5′-TGAGTCCTGGGCAGAAGTTT-3′	
**GAPDH**	sense	5′- AGGTGACACTATAGAATAAA GGTGAAGGTCGGAGT CAA -3′	68.0°C
	anti-sense	5′- GTACGACTCACTATAGGGAGA TCTCGCTCCTGGAAGATG -3′	
**BCR/ABL (b3a2)**	sense	5′- GCATTCCGCTGACCATCAATA -3′	58.5°C
	anti-sense	5′- TCCAACGAGCGGCTTCAC -3′	

### Cellular surface expression of ABCG2

For the phenotypic analysis of the cell lines, the cells were washed in PBS and then stained for 30 min at room temperature with a PE anti-human CD338 antibody (ABCG2, 1∶100, Biolegend, San Diego, CA, USA), and a PE-IgG2b isotype control (1∶100, Biolegend, San Diego, CA, USA) used as a negative control. Finally, the cells were washed twice with ice-cold PBS and then analyzed by flow cytometry (Becton Dickinson, Mountain View, CA, USA).

### Side population analysis

The cell suspensions were labeled with Hoechst 33342 (Invitrogen, Carlsbad, CA, USA) dye for side population analysis according to standard protocol [23]. Cells were briefly resuspended in pre-warmed Hepes buffer containing 2% FBS at a density of 10^6^/ml. Hoechst 33342 dye (Invitrogen, Carlsbad, CA, USA) was then added at a final concentration of 5 µg/ml (37°C for 90 min) in the presence or absence of verapamil (50 µM; Sigma) with intermittent shaking. The cells were counterstained with 2 µg/ml propidium iodide (PI) (Sigma–Aldrich) to label dead cells and were then analyzed using a FACS Vantage SE cell sorter (Becton Dickinson, Mountain View, CA, USA) by a dual-wavelength analysis (450/20 and 675/20 nm) after excitation at 350 nm.

### Small interfering RNA transfection

Synthetic control small interfering RNA (siRNA) and siRNA against PTEN were purchased from Santa Cruz Biotechnology. Approximately 2×10^5^ cells were seeded in 6-well culture plates and incubated for 72 h prior to transfected with 80 nM siRNA using Lipofectamine 2000 (Invitrogen Corp., Carlsbad, CA, USA) as directed by the manufacturer.

### Statistical analysis

The comparisons of the chronic myeloid leukemia patients and normal donors or among the cell lines were made using the Statistical Package for the Social Sciences (SPSS) version 17.0. Differences were considered significant for *P*<0.05. The differences between the populations were calculated using Student's t test or one-way ANOVA, as appropriate. The diagrams were created using GraphPad Prism 5 software.

## Results

### ABCG2 overexpression decreased drug sensitivity and drug-induced inhibition of DNA synthesis in leukemia cell lines

**Figure 1:**
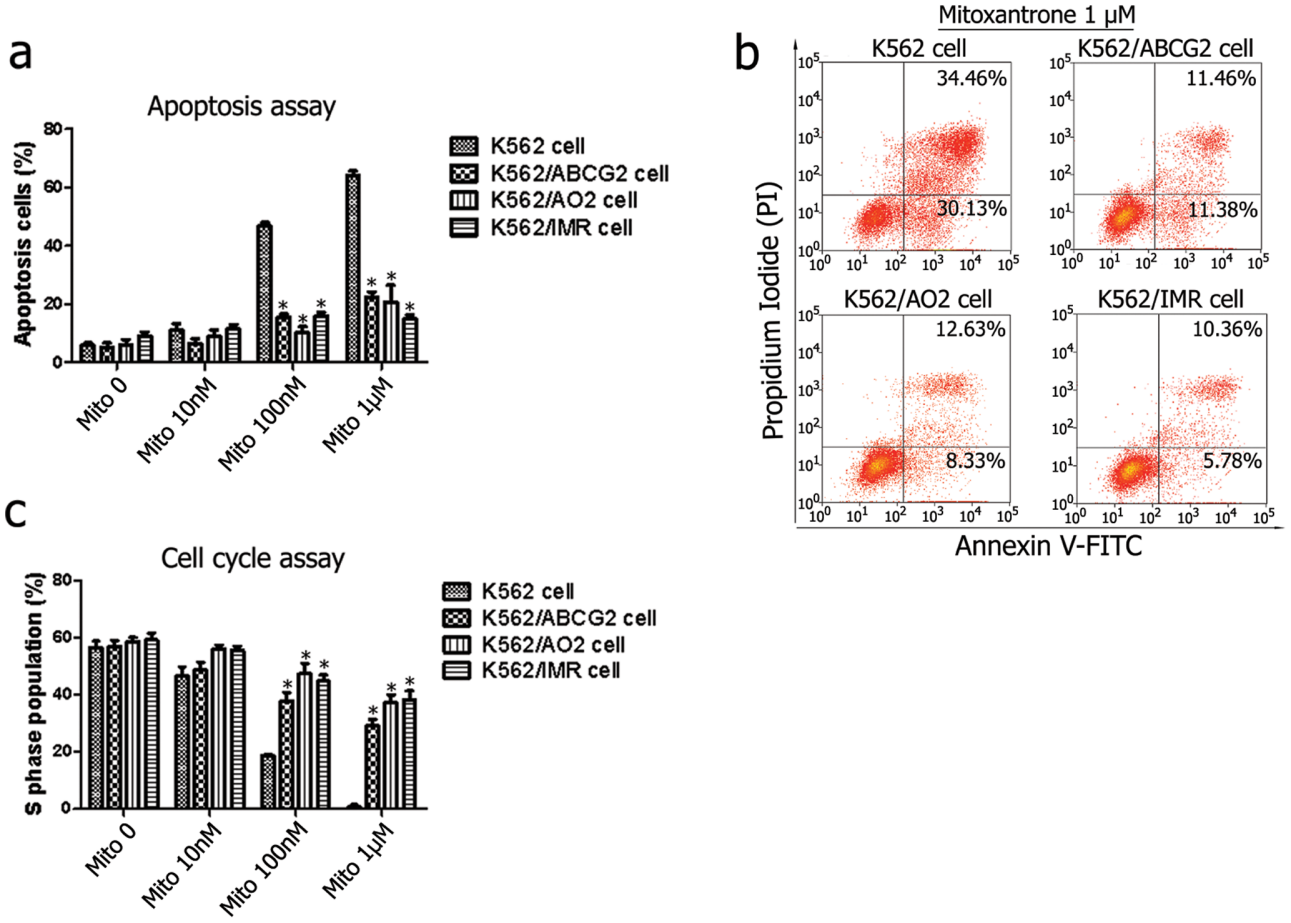
K562 cells with ABCG2 overexpression exhibited attenuated apoptosis and were no longer arrested in S phase by mitoxantone treatment. (**a**, **b**) After exposure to 10 nM, 100 nM or 1 µM of mitoxantone for 72 h, the cell lines were subjected to flow cytometry to quantify the number of apoptotic cells, and an increased drug resistance was observed in K562/ABCG2, K562/AO2 and K562/IMR cells compared with wild-type K562 cells. (**c**) The cell cycle phase was determined by flow cytometry after treatment with different concentrations of mitoxantone, which decreased the inhibition of DNA synthesis at S phase in K562/ABCG2, K562/AO2 and K562/IMR cells compared with wild-type K562 cells. The histogram represented the means ± s.d. for three replicates. *P<0.05.

Cellular resistance to imatinib can arise through a variety of mechanisms, including mutations in the BCR-ABL kinase and increased efflux by multidrug resistance transporters such as P-glycoprotein (P-gp) and ABCG2 [24], [25], [26]. Imatinib stimulates ABCG2-specific ATPase activity and is both a substrate and an inhibitor of ABCG2 [13], [27]. To determine whether ABCG2 is related to CML resistance, the K562, K562/ABCG2 (ABCG2 overexpressing), K562/AO2 (resistance to adriamycin) and K562/IMR (resistance to imatinib) cell lines were exposed to 10 nM, 100 nM or 1 µM of mitoxantone. Compared with the wild-type K562 cells, increased mitoxantrone resistance was observed in K562/ABCG2, K562/AO2 and K562/IMR cells after 72-h treatment with 100 nM or 1 µM of mitoxantone (*P*<0.05) (Fig. 1a and Table 3), with comparable mitoxantrone-induced apoptosis (*P*>0.05) (Fig. 1b). The results indicated that ABCG2 might attenuate the cytotoxic effects of mitoxantrone in CML.

**Table 3:** The ratio of viable cells after 72-h treatment with mitoxantone at a range of concentrations in the presence of LY294002 or rapamycin by CKK8 assay.

	K562			K562/ABCG2			K562/AO2			K562/IMR		
Mitoxantrone	−	+LY	+Rapa	−	+LY	+Rapa	−	+LY	+Rapa	−	+LY	+Rapa
**10 nM**	0.90±	0.80±	0.80±	0.91±	0.81±	0.79±	0.91±	0.85±	0.82±	0.91±	0.83±	0.79±
	0.04	0.07	0.07	0.04	0.08	0.08	0.05	0.04	0.06	0.06	0.07	0.09
**100 nM**	0.60±	0.48±	0.53±	0.82±	0.58±	0.52±	0.85±	0.76±	0.69±	0.53±	0.50±	0.50±
	0.08	0.07‡	0.07§	0.06†	0.07* ‡	0.04* §	0.05†	0.07	0.07	0.05†	0.08* ‡	0.09* §
**1 µM**	0.39±	0.17±	0.23±	0.72±	0.24±	0.23±	0.80±	0.43±	0.38±	0.83±	0.22±	0.19±
	0.05	0.05* ‡	0.06* §	0.08†	0.07* ‡	0.04* §	0.07†	0.06*	0.06*	0.08†	0.08* ‡	0.06* §

Each value represents the mean value ± s.d. from three individual experiments.

* Versus cells without LY294002 or rapamycin treatment within each cell group, *P*<0.05.

† Versus K562 wild-type cells without LY294002 or rapamycin when treated with the same concentration of mitoxantrone, *P*<0.05.

‡ Versus K562/AO2 cells among all of the experimental cells treated with LY294002, *P*<0.05.

§ Versus K562/AO2 cells among all the experimented cells treated with rapamycin, *P*<0.05.

Next, we evaluated the effect of ABCG2 overexpression on the mitoxantrone-induced cell cycle changes in leukemia cell lines. As shown in Fig. 1c and Fig. S4, after 72-h treatment with mitoxantrone, the proportion of K562, K562/ABCG2, and K562/AO2 cells in S phase was significantly decreased in concentration-dependent manner (*P*<0.05), indicating an inhibition in DNA synthesis by mitoxantrone. However, compared with wild-type K562 cells, the subpopulation in the S phase was remarkably higher in K562/ABCG2, K562/AO2 and K562/IMR cells when treated with 100 nM or 1 µM of mitoxantone (*P*<0.05). These findings suggest that S-phase blockage might be an underlying mechanism contributing to drug resistance and the pivotal cross between drug resistance and tumor growth.

### ABCG2 expression regulated the SP phenotype, and PTEN protein deletion increased p-Akt in resistant leukemia cells

**Figure 2:**
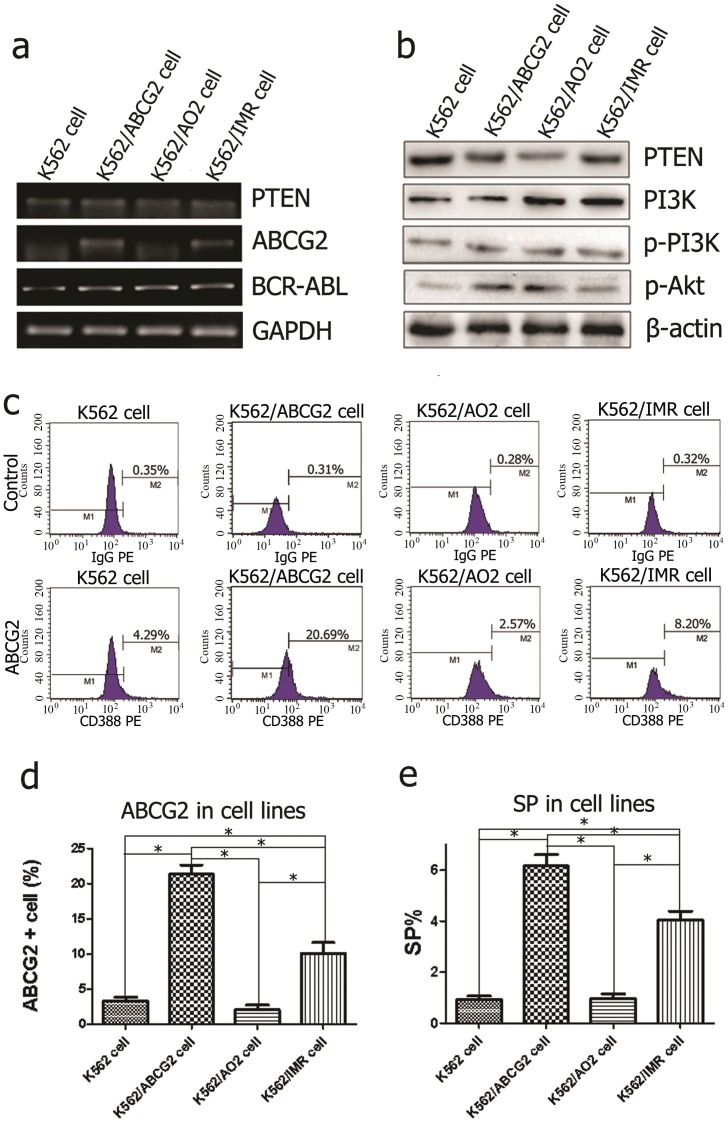
Detection of the PTEN/PI3K/Akt signal pathway, ABCG2 and SP in leukemia cells. (**a**) BCR-ABL transcript expression was upregulated in all drug-resistant cells, whereas higher ABCG2 transcript was only detected in K562/ABCG2 and K562/IMR cells. (**b**) Western blot analysis revealed decreased PTEN protein but increased p-Akt expression in K562/ABCG2, K562/AO2 and K562/IMR cells. (**c**) Expression of CD338 (anti-ABCG2) on the cell surface were detected by FCM. IgG2b-PE was used as an isotype control. (**d**) The distribution of ABCG2+ cells was detected by flow cytometry (FCM). The histogram demonstrates a higher ratio of ABCG2+ cells in K562/ABCG2 and K562/IMR cell lines. The results were represented as the mean ± s.d. of three experiments. *P<0.05. (**e**) The increased SP fraction was observed in K562/ABCG2 and K562/IMR cells. Each sample was incubated with 50 µM verapamil as a control, and only PI-negative (live) cells were gated to be analyzed.

As reported, PTEN deletion influences the disease progression of both CML and B-ALL by regulating its downstream target Akt1 [21], through which the BCR-ABL fusion gene regulates ABCG2 expression [28]. Thereby, we evaluated the endogenous levels of PTEN, PI3K, p-PI3K, p-Akt and ABCG2 in four leukemia cell lines. Compared with K562 cells, the expression level of PTEN protein was decreased significantly in all drug-resistant cells, with upregulated levels of BCR-ABL transcript detected by RT-PCR (Fig. 2a) (Tab. 2) and upregulated p-Akt protein detected by western blot (Fig. 2b). Additionally, ABCG2 mRNA levels (Fig. 2a) were significantly higher and the ABCG2+ population (Fig. 2c and d) was significantly larger in K562/ABCG2 and K562/IMR cells than in K562 and K562/AO2 cells. The siRNA-mediated knockdown of PTEN significantly increased ABCG2 transcript level in K562/ABCG2 cell (Fig. S3). These results suggested that the down-regulation of PTEN along with both p-Akt and ABCG2 upregulation might contribute to drug resistance in K562/ABCG2 and K562/IMR cells but not in K562/AO2 cells.

Because functional ABCG2 has been reported to be overexpressed on the surface of primary CML stem cells [29], we combined surface marker characteristics with Hoechst dye efflux to explore the interaction of the PI3K/Akt pathway, ABCG2 expression and the SP fraction specifically in the primitive stem cell subset. Flow cytometry confirmed higher fractions of SP cells in the K562/ABCG2 and K562/IMR cell lines compared with K562 and K562/AO2 cells (*P*<0.05) (Fig. 2e and Fig. S1). In addition, ABCG2 transcript levels were quantified by RT-PCR in Hoechst 33342-labeled sorted SP and non-SP fractions. Higher expression level of ABCG2 was present in the SP fraction compared to the non-SP fraction in K562/ABCG2 cell, whereas no similar response was yielded in K562 cell (Fig. S2a–b) These findings further demonstrated that ABCG2 might contribute essentially to the SP phenotype.

### PTEN regulated the ABCG2+ fraction, SP phenotype and drug sensitivity through the PI3K/Akt pathway in leukemia cells

**Figure 3:**
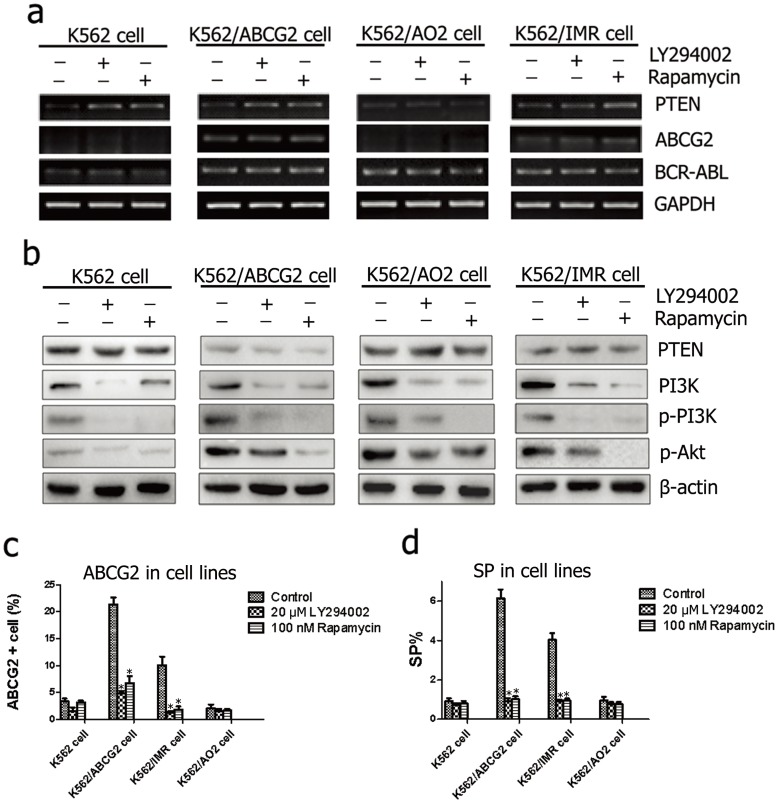
PI3K/Akt pathway activation and the fraction of ABCG2+ and SP cells was detected by RT-PCR, western blotting and FACS analysis in leukemia cells before or after incubation with 20 µM LY294002 or 100 nM rapamycin for 72 h. (**a, b**) After LY294002 or rapamycin treatment, activation of the PTEN transcript and down-regulation of p-PI3K and p-Akt were detected in four cell lines. (**c**) A reduced fraction of ABCG2 cells in K562/ABCG2 and K562/IMR cells was observed after treatment. (**d**) The increased SP fraction was abolished by LY294002 or rapamycin treatment in K562/ABCG2 and K562/IMR cells. All results are presented as the means ± s.d. from three independent experiments. *P<0.05.

To determine whether the PI3K/Akt pathway is involved in drug resistance through the regulation of ABCG2, leukemia cells were treated with the PI3K inhibitor LY294002 (20 µM) or the mTOR inhibitor rapamycin (100 nM) for 72 h. Although the mRNA levels of both ABCG2 and BCR-ABL did not significantly change (Fig. 3a), activation of PTEN transcript and the down-regulation of both p-PI3K and p-Akt (Fig. 3b), as well as a reduced proportion of ABCG2+ cells (Fig. 3c), were observed in treated K562/ABCG2 and K562/IMR cells, suggesting that PI3K and Akt activity might regulate ABCG2 expression. Furthermore, we observed that incubation with LY294002 or rapamycin decreased the fractions of SP cells in K562/ABCG2 and K562/IMR cells (*P*<0.05) (Fig. 3d and Fig. S1). Taken together, these results indicated that the PI3K/Akt pathway participated in regulating the SP fraction through ABCG2.

Furthermore, as shown in Table 3, CCK8 analysis demonstrated that the incubation of leukemia cells with LY294002 or rapamycin strongly increased the sensitivity of leukemia cells to 100 nM or 1 µM of mitoxantrone (*P*<0.05), suggesting that chemosensitivity to mitoxantrone is tightly correlated with decreased p-PI3K and p-Akt expression in the resistant K562 cells. When pretreated with either LY294002 or rapamycin, K562/ABCG2 and K562/IMR cells exhibited a more significant decrease in the proportion of viable cells after treatment with 100 nM or 1 µM mitoxantrone than did K562/AO2 cells (*P*<0.05). These data suggest that p-PI3K, p-Akt or ABCG2 activity may fractionally participate in chemoresistance in K562/AO2 cells.

### No substantial difference in the ABCG2+ fraction but an increased SP fraction in CML patients in AP/BP compared with the control group

**Figure 4:**
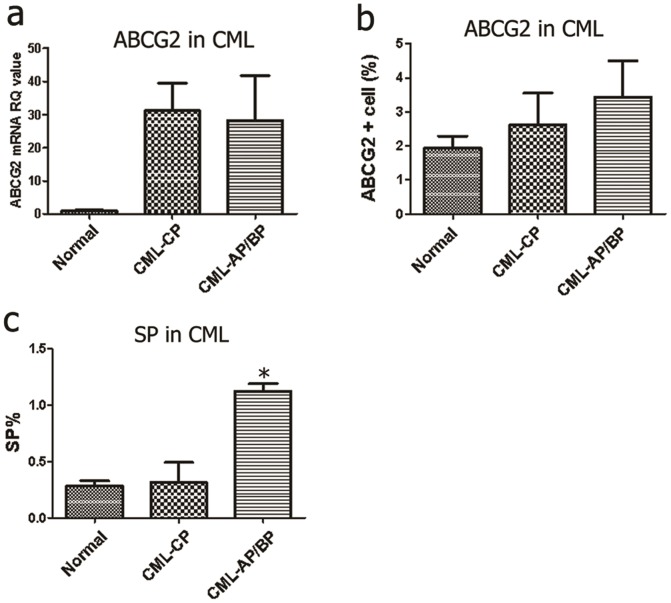
Endogenous expression of ABCG2 and SP fractions in CML patients. (**a**) The ABCG2 transcript in CML patients was determined by real-time RT-PCR using the RQ values with *GAPDH* mRNA as the endogenous control. In contrast to normal donors, no substantial difference in the ABCG2 transcript levels was detected in CML patients. *P<0.05. (**b**) ABCG2+ cells were labeled by CD338-PE and detected by FACS, using IgG2b-PE as the isotype control. There were no significant differences among the groups. *P<0.05. (c) Significantly higher percentages of SP cells were observed in CML blasts at AP/BP using flow cytometry. *P<0.05.

Based on the results of CML cell lines, the endogenous mRNA and protein levels of *ABCG2* in 61 consecutive CML patients with Ph-positive metaphases and/or BCR-ABL–positive transcripts in the CP (chronic phase) and in 35 patients in AP/BP (accelerated phase/blastic phase) were detected by real-time RT-PCR and FACS analysis, respectively, at our hospital (Tab. 1). ABCG2+ cells and the mRNA levels in CML patients in different phases exhibited no substantial differences (P>0.05) but were obviously higher than in normal donors (Fig. 4a–b).

Stem cells are frequently identified as the “side population” by flow cytometry based on ABCG2-mediated efflux of Hoechst 33342 dye. Incubation of the leukemia cells with 50 µM verapamil, known to block ABC transporter activity, abolished part of the SP as a negative control. ABCG2 transcript differed significantly between SP and non-SP cells in some CML patient (Fig. S2c–d). Flow cytometry revealed a very small SP fraction, ranging from 0.11% to 1.14% in the cases, and a significantly higher percentage of the SP cells in CML patients at AP/BP compared with the healthy donors (*P*<0.05) (Fig. 4c). Our data suggest that an increase in the SP fraction might confer a survival advantage to CML cells.

**Figure 5:**
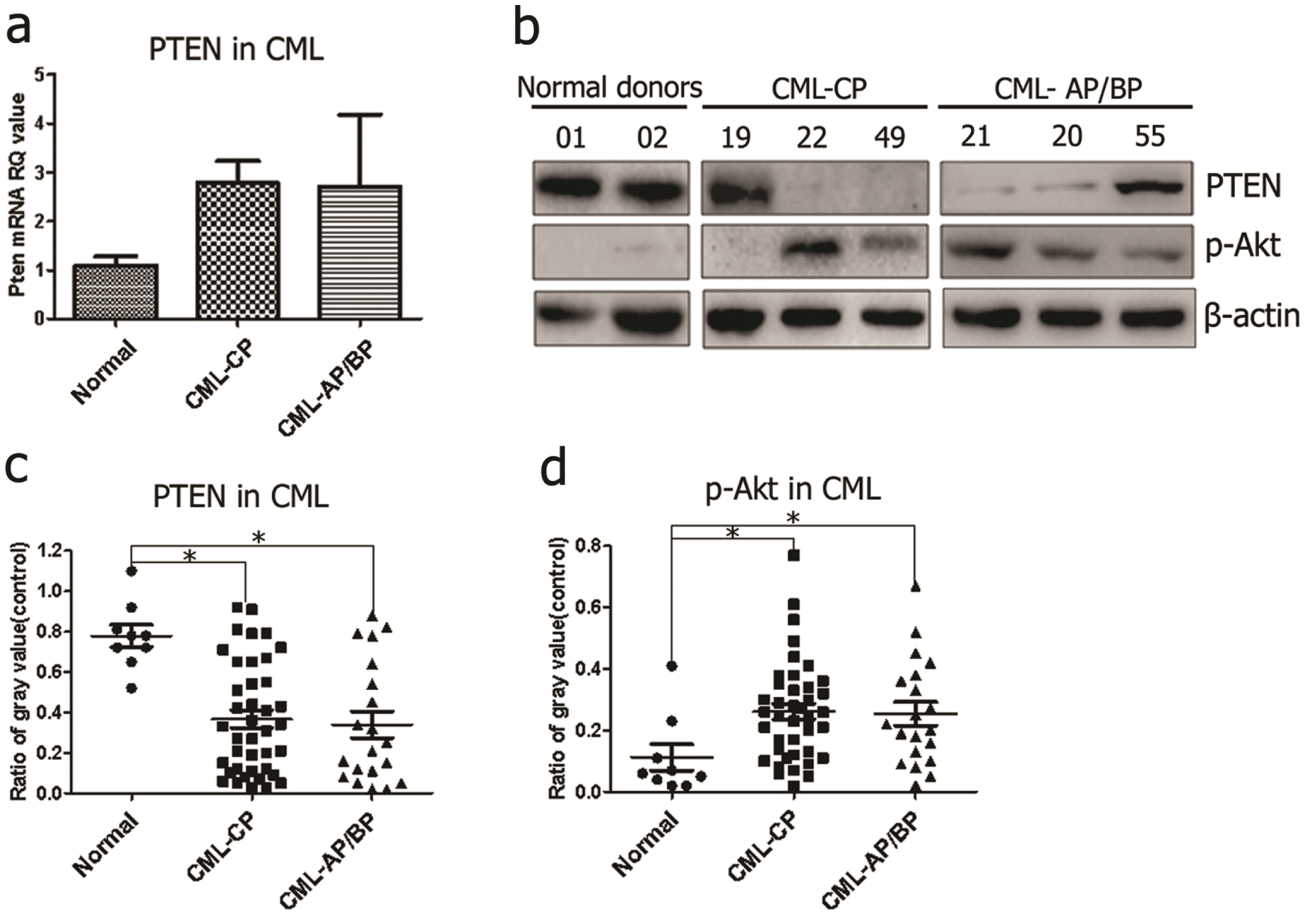
Endogenous expression of the PTEN/PI3K/Akt pathway in CML patients. (**a**) Endogenous levels of *PTEN* mRNA in CML patients and normal donors were examined by real-time RT-PCR. *PTEN* transcript was not notably decreased in leukemia blasts compared with the normal donors. *P<0.05. (b–d) These results suggested that p-Akt might be negatively regulated by PTEN in CML blasts by western blot analysis. The ratio of the gray value was calculated by band intensities using ImageJ software (Wayne Rasband, NIH). β-actin was used as a control for protein loading. All histograms represent the mean of one experiment performed in triplicate ± s.d.

The SP phenotype was correlated with low PTEN and high p-Akt levels in CML patients. PTEN maintains normal hematopoietic stem cells and prevents leukemia development from leukemia stem cells [30], [31]. To determine whether increased ratios of the SP would be related to the down-regulation of PTEN, both transcript and protein levels of PTEN were detected in CML patients. In leukemia blasts, the levels of PTEN transcript were surprisingly increased compared to the normal donors (*P*>0.05) (Fig. 5a). However, PTEN protein was remarkably decreased in CML patients in AP/BP compared with other patients (P<0.05), suggesting that low PTEN protein accompanying the SP phenotype was limited to the status of the disease. Furthermore, p-Akt was activated in some CML samples with low expression of PTEN protein (P<0.05) (Fig. 5b–d). Our study further demonstrated that the SP fraction may contribute to the progression of CML, as indicated by decreased PTEN protein expression and Akt activation.

## Discussion

In light of the crucial role of the BCR-ABL tyrosine kinase in chronic myelogenous leukemia, TKIs have become the first-line therapy for most patients with chronic myelogenous leukemia [4], [32],[33]. However, TKIs do not kill CML stem cells [34]. Although more than 80% of CML patients in chronic phase can achieve an ongoing complete hematologic response after treatment with imatinib, a considerable number of cases eventually progress to the accelerated phase and even blast crisis [35], [36]. Additionally, BCR-ABL-positive malignant cell clones have been shown to persist within the CD34+ stem cell fractions, even in CML patients for whom imatinib had induced a complete cytogenetic remission [37]. Given that CML patients harbor quiescent CML stem cells that may serve as reservoir for disease progression to blast crisis, there is a strong possibility of the existence of imatinib-refractory CML stem cells.

The drug pump ABCG2 is a multidrug resistance protein and is well known as a specific phenotype of the SP cells with stem-like properties [15]. Consistent with previous reports that imatinib is an inhibitor and substrate for ABCG2 [13],[27],[28], we observed upregulation of ABCG2 in K562/IMR cells and K562/ABCG2 cells, both of which accordingly exhibited higher ratios of SP cells and decreased susceptibility to mitoxantrone due to higher levels of ABCG2. Furthermore, PTEN was revealed to be involved in ABCG2-mediated multi-drug resistance for CML through the PI3K/Akt signaling pathway in our study. Lower PTEN expression was observed in drug-resistant CML cells; consistent with our reports in human embryonic stem cells, the over-expression of ABCG2 in H9 cells leads to p-Akt activation [38]. Higher p-Akt expression levels were also detected in both K562/ABCG2 and K562/IMR cells. Second, consistent with previous studies in human glioma, primary esophageal carcinoma and epithelial carcinoma cells [22], [39], [40], our data demonstrated increased drug sensitivity, down-regulation of p-PI3K and p-Akt, suppression of the ABCG2 and a decrease in the SP fraction in K562/ABCG2 and K562/IMR cells after LY294002 or rapamycin treatment. These findings suggest that the chemotherapeutic sensitivity and the fractions of ABCG2 and SP cells in drug-resistant CML might be mediated by the PI3K/Akt signaling pathway, which is consistent with our previous reports in acute leukemia [41]. Therefore, accumulating data provide underlying connections among the PTEN, PI3K/Akt pathway, multidrug resistance transporters, stem-like character, and therapeutic resistance, suggesting that activation of this pathway also enhances the ability of CML cancer stem-like cells to expel drugs.

This study further focused on the complex interaction between PTEN and ABCG2 in CML cases to explore whether such regulation existed in the clinic process. Inconsistent with data from CML cell lines, no substantial differences in the ABCG2+ phenotype and ABCG2 mRNA levels were detected among different stages of CML blasts and normal cells. Additionally, the research in glioma tumor reported that PI3K inhibitor treatment changed the activity of ABCG2 in neurospheres, but the expression levels of the mRNA and protein were unaffected [22]. These data indicate that ABCG2 function, rather than its mRNA or protein expression, might play a more important role in initiation and progress of CML. SP cells are also found in a variety of mammalian species, including humans, where their frequency is low [42]. Inconsistent with ABCG2 expression, a higher percentage of SP compared with the donors was observed in the AP/BP group, which partly suggested that monitoring the SP ratio could predict disease progression and might be an optimal indicator to represent in vivo ABCG2 function. Meanwhile, similar to the results from CML cell lines, this study also demonstrated that absent/low expression of PTEN at the protein level and subsequent p-Akt activation in the CML groups might promote the acceleration of CML development and an increased SP ratio. Nevertheless, more efforts are needed to reveal the precise mechanism of how loss of the tumor suppressor PTEN regulates ABCG2 function and further enhances the SP phenotype through the PI3K/Akt pathway in CML.

In summary, to investigate the perplexing relationships among PTEN, ABCG2 and the SP in CML, our studies demonstrated that PTEN played an essential role in regulating the SP in CML through the PI3K/Akt signaling pathway *in vitro*. Then, our studied revealed that the SP phenotype and ABCG2 function rather than ABCG2 expression was correlated with drug resistance and disease progression in CML patients, which was mediated at least partially by p-Akt activation. Therefore, intervention in the functional enhancement of p-Akt mediated by the loss of PTEN inhibition would provide a potential therapeutic strategy for targeting CML stem cells.

## Supporting Information

Figure S1 **The distribution of the SP phenotype was assessed by flow cytometry in cell lines before and after treatment with LY294002 or rapamycin.** Each sample was incubated with 50 µM verapamil as a control, and only PI-negative (live) cells were gated to be analyzed. The ABCG2+ population was significantly larger in K562/ABCG2 and K562/IMR cells than in the other cell types.
(TIF)

Figure S2 **ABCG2 transcript in the SP fraction.** ABCG2 mRNA was analyzed by RT-PCR in the flow cytometry-selected SP fraction and compared with the non-SP fraction in K562 cells (**a**), K562/ABCG2 cells (**b**), CML-CP patient No. 23 (**c**) and CML-AP/BP patient No. 9 (**d**). GAPDH was used as a control.
(TIF)

Figure S3 **siRNA directed against PTEN specifically inhibited PTEN expression in leukemia cell lines.** One hundred nanomolar siRNA directed against PTEN specifically inhibited PTEN expression in the K562/ABCG2 cell line. RT-PCR was performed 48 h after the leukemia cells were treated with PTEN siRNA or control siRNA to evaluate PTEN and ABCG2 expression.
(TIF)

Figure S4 **K562 cells overexpressing ABCG2 overcame mitoxantrone-induced S-phase arrest.** (**a**, **b**) After exposed to 10 nM, 100 nM or 1 µM of mitoxantone for 72 h, the cell lines were subjected to flow cytometry to determine the cell cycle distribution, and a decreased the inhibition of DNA synthesis at S phase was observed in the K562/ABCG2, K562/AO2 and K562/IMR cells compared with wild-type K562 cells. The histogram represented the means ± s.d. for three replicate determinations. *P<0.05.
(TIF)
