# Correction: PTEN Regulates BCRP/ABCG2 and the Side Population through the PI3K/Akt Pathway in Chronic Myeloid Leukemia

**DOI:** 10.1371/journal.pone.0099917

**Published:** 2014-06-05

**Authors:** The PLOS ONE Staff

The authors are listed out of order. Please view the correct author order, affiliations, and citation here:

Fang-Fang Huang^1^, Li Zhang^2^, Deng-Shu Wu^1^, Xiao-Yu Yuan^1^, Yan-Hui Yu^1^, Xie-Lan Zhao^1^, Fang-Ping Chen^1^, Hui Zeng^1^

1 Department of Hematology, Xiang Ya Hospital, Central South University, Changsha, Hunan, China

2 Department of Hematology, West China Hospital, Si Chuan University, Chengdu, Sichuan, China

Huang F-F, Zhang L, Wu D-S, Yuan X-Y, Yu Y-H, et al. (2014) PTEN Regulates BCRP/ABCG2 and the Side Population through the PI3K/Akt Pathway in Chronic Myeloid Leukemia. PLoS ONE 9(3): e88298. doi:10.1371/journal.pone.0088298

## References

[1] Huang F-F, Zhang L, Wu D-S, Yuan X-Y, Chen F-P, et al (2014) PTEN Regulates BCRP/ABCG2 and the Side Population through the PI3K/Akt Pathway in Chronic Myeloid Leukemia. PLoS ONE 9(3): e88298 doi:10.1371/journal.pone.0088298 24603487 10.1371/journal.pone.0088298 PMC3945754

