# Supplementary figures and images for: PTEN Regulates BCRP/ABCG2 and the Side Population through the PI3K/Akt Pathway in Chronic Myeloid Leukemia
Source: PLoS One. 2014 Mar 6;9(3):e88298. doi: 10.1371/journal.pone.0088298 (PMC3945754; doi:10.1371/journal.pone.0088298)

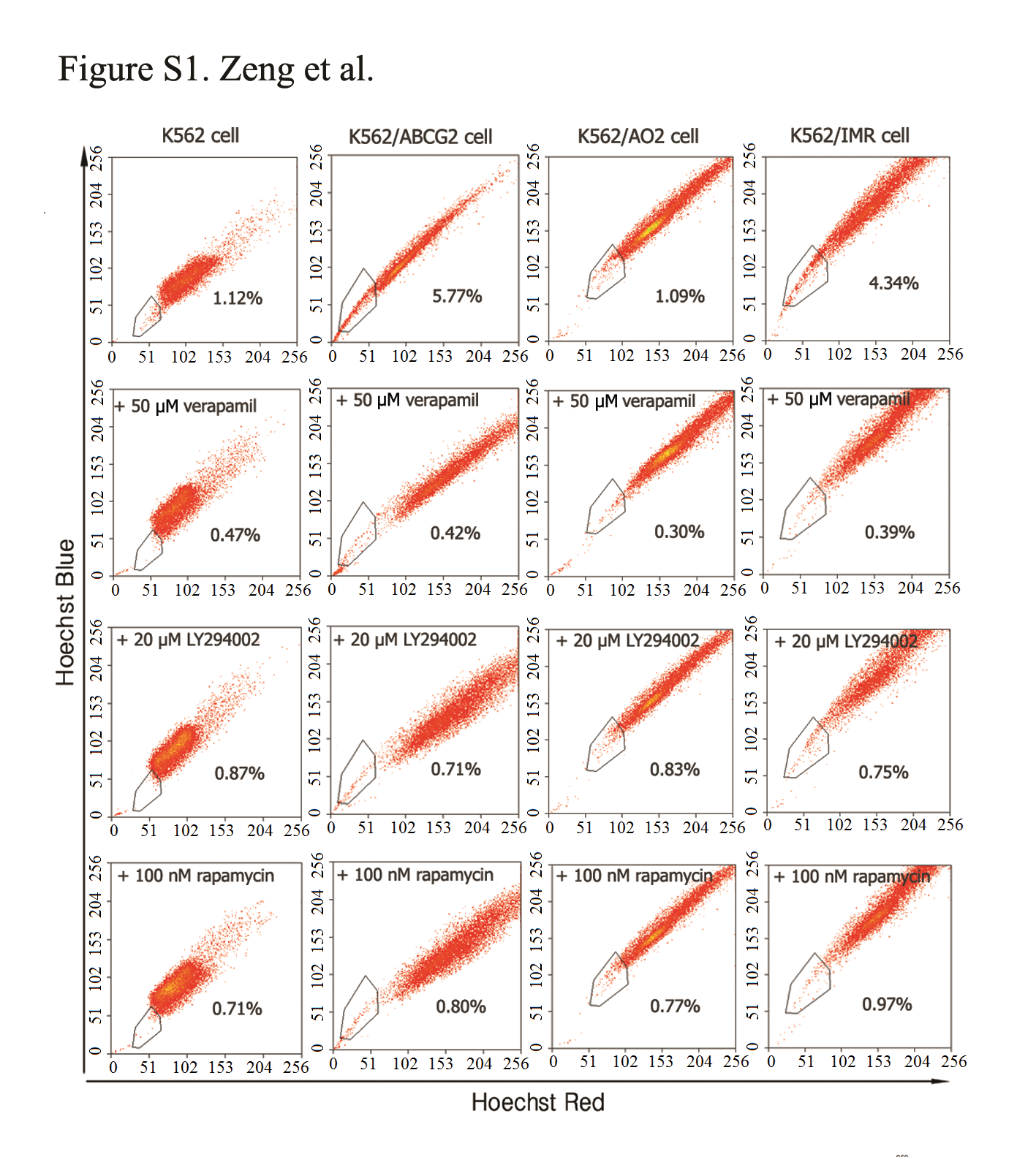

Supplement: Figure S1 — The distribution of the SP phenotype was assessed by flow cytometry in cell lines before and after treatment with LY294002 or rapamycin. Each sample was incubated with 50 µM verapamil as a control, and only PI-negative (live) cells were gated to be analyzed. The ABCG2+ population was significantly larger in K562/ABCG2 and K562/IMR cells than in the other cell types. (TIF) [file pone.0088298.s001.tif]

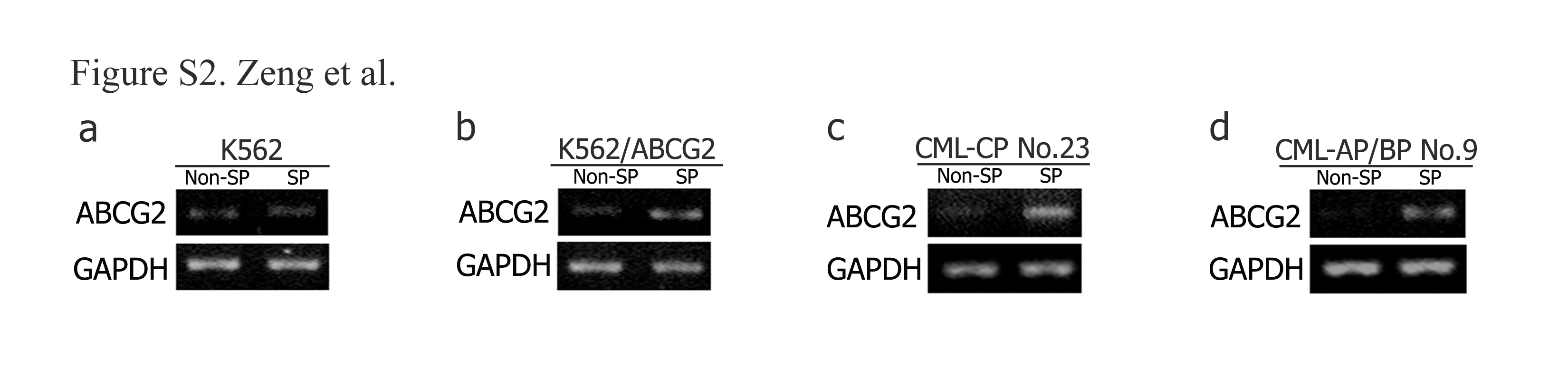

Supplement: Figure S2 — ABCG2 transcript in the SP fraction. ABCG2 mRNA was analyzed by RT-PCR in the flow cytometry-selected SP fraction and compared with the non-SP fraction in K562 cells (a), K562/ABCG2 cells (b), CML-CP patient No. 23 (c) and CML-AP/BP patient No. 9 (d). GAPDH was used as a control. (TIF) [file pone.0088298.s002.tif]

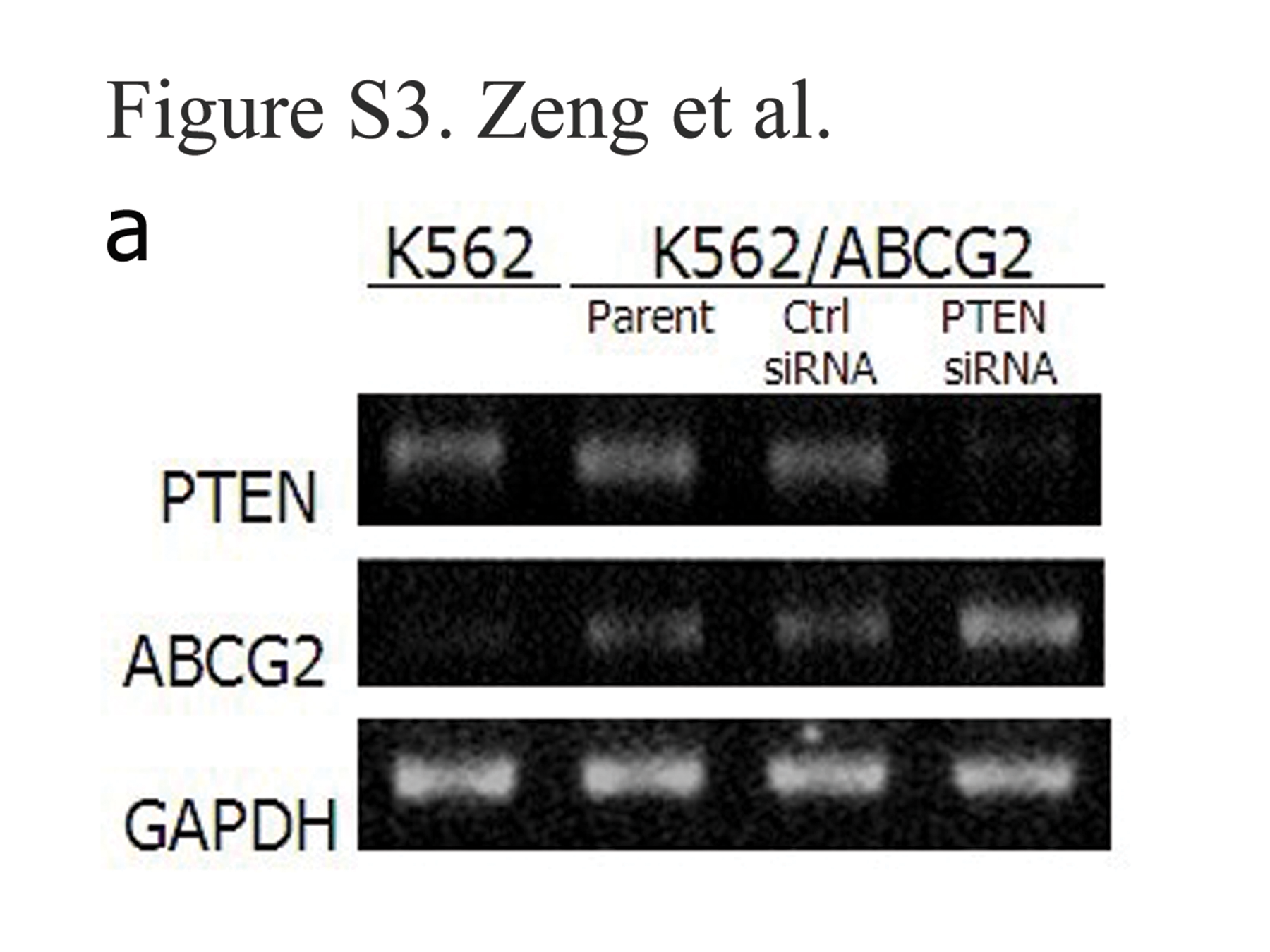

Supplement: Figure S3 — siRNA directed against PTEN specifically inhibited PTEN expression in leukemia cell lines. One hundred nanomolar siRNA directed against PTEN specifically inhibited PTEN expression in the K562/ABCG2 cell line. RT-PCR was performed 48 h after the leukemia cells were treated with PTEN siRNA or control siRNA to evaluate PTEN and ABCG2 expression. (TIF) [file pone.0088298.s003.tif]

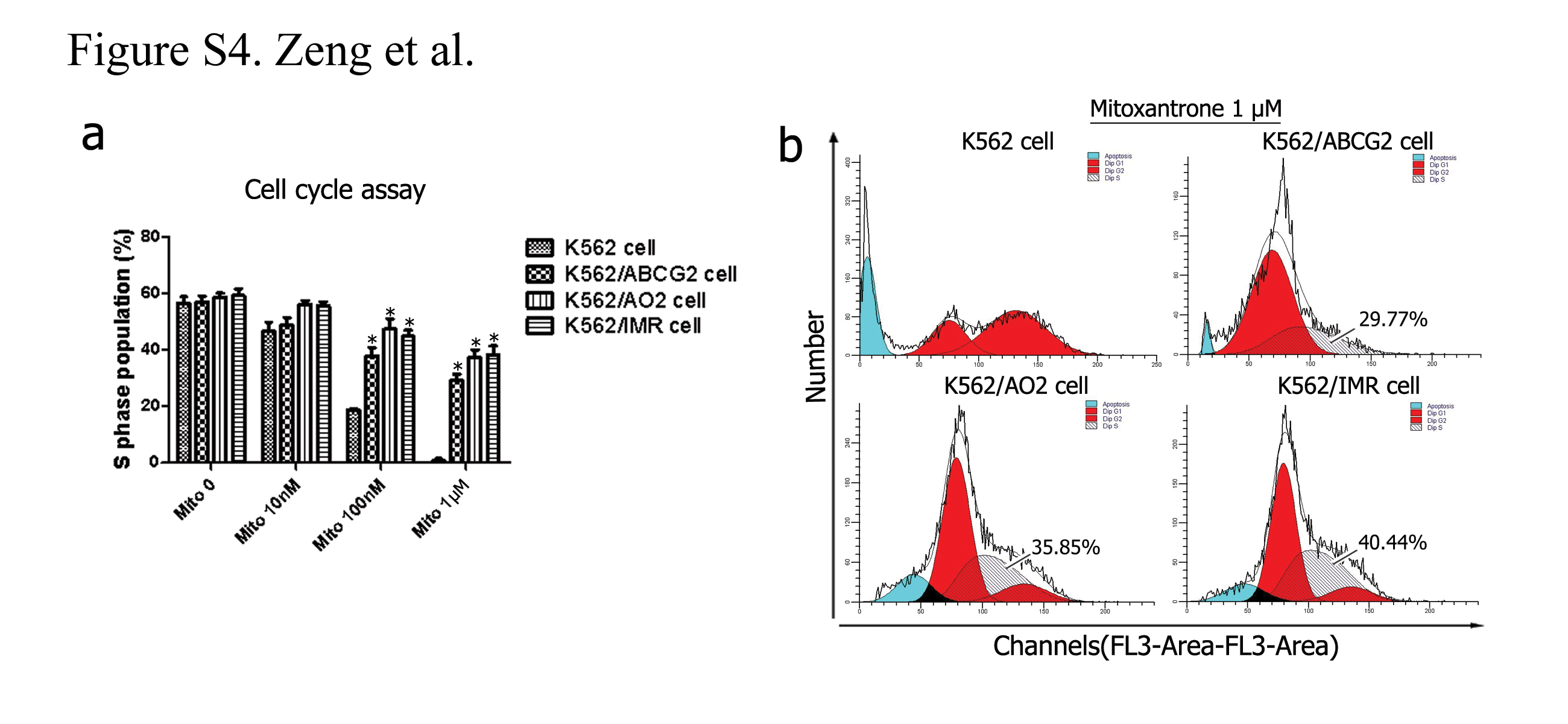

Supplement: Figure S4 — K562 cells overexpressing ABCG2 overcame mitoxantrone-induced S-phase arrest. (a, b) After exposed to 10 nM, 100 nM or 1 µM of mitoxantone for 72 h, the cell lines were subjected to flow cytometry to determine the cell cycle distribution, and a decreased the inhibition of DNA synthesis at S phase was observed in the K562/ABCG2, K562/AO2 and K562/IMR cells compared with wild-type K562 cells. The histogram represented the means ± s.d. for three replicate determinations. *P<0.05. (TIF) [file pone.0088298.s004.tif]
